# The ginsenoside Rk3 exerts anti-esophageal cancer activity *in vitro* and *in vivo* by mediating apoptosis and autophagy through regulation of the PI3K/Akt/mTOR pathway

**DOI:** 10.1371/journal.pone.0216759

**Published:** 2019-05-15

**Authors:** Huanhuan Liu, Jiaqi Zhao, Rongzhan Fu, Chenhui Zhu, Daidi Fan

**Affiliations:** 1 Shaanxi Key Laboratory of Degradable Biomedical Materials, School of Chemical Engineering, Northwest University, Xi’an, China; 2 Shaanxi R&D Center of Biomaterials and Fermentation Engineering, School of Chemical Engineering, Northwest University, Xi’an, China; 3 Biotech.&Biomed, Research Institute, Northwest University, Xi’an, China; 4 School of Pharmaceutical Sciences, Guangzhou University of Chinese Medicine, Guangzhou, China; Institute of Biochemistry and Biotechnology, TAIWAN

## Abstract

The rare ginsenoside Rk3 is a bioactive component derived from ginseng and *Panax notoginseng* that has been proven to possess anti-lung cancer activity. However, the effect of Rk3 on human esophageal cancer has not yet been reported. In this study, we aimed to explore its anticancer curative effect and potential molecular mechanisms in the Eca109 and KYSE150 cell lines. We found that Rk3 was able to significantly repress cell proliferation and colony formation in both Eca109 and KYSE150 cells *in vitro*. In the KYSE150 xenograft model, Rk3 obviously inhibited tumor growth and exhibited little toxicity in organs. Moreover, Rk3 could trigger G1 phase arrest and induce apoptosis and autophagy. Interestingly, apoptosis induced by Rk3 could be partly abrogated by 3-MA (an autophagy inhibitor), implying that autophagy could enhance apoptosis. Further studies indicated that pretreatment with the Akt inhibitor GSK690693 or the mTOR inhibitor rapamycin promoted Rk3-induced apoptosis and autophagy, demonstrating that the PI3K/Akt/mTOR pathway is related to Rk3-induced apoptosis and autophagy. In conclusion, the present study is the first to clarify that Rk3 can inhibit Eca109 and KYSE150 cell proliferation through activating apoptosis and autophagy by blocking the PI3K/Akt/mTOR pathway, suggesting that Rk3 may be a promising antitumor agent for esophageal cancer. In addition, this study provides ideas and an experimental basis for further research on the anti-esophageal cancer effects of the ginsenoside Rk3 and its mechanism.

## Introduction

Human esophageal cancer is one of the most aggressive malignancies worldwide, and the esophageal squamous cell carcinoma accounts for more than 90% of all cases of this type of cancer [1, 2]. Although there has made considerable progress in early screening and surgery combined with radiotherapy or chemotherapy, the prognosis for esophageal cancer patients remains poor, with an overall five-year survival rate of less than 30% [3, 4]. At present, the most common treatment for many esophageal cancer patients is chemotherapy. Cis-platinum, a platinum-containing chemotherapy drug, is considered the most effective agent for the treatment of esophageal cancer. Unfortunately, this drug causes a range of dose-limiting toxicities, such as renal toxicity, liver toxicity, neutropenia, myelosuppression and drug resistance [5, 6]. Therefore, there is an urgent need to obtain new effective antineoplastic drugs with fewer adverse effects.

At present, traditional Chinese medicine (TCM) has attracted much attention because of its advantages of reducing the side effects and drug resistance of anticancer chemotherapy. Ginsenosides are a main bioactive components derived from ginseng and *Panax notoginseng* and possess multiple biological activities, such as antiinflammatory, antioxidative, and antitumor effects [7, 8]. The ginsenoside Rg3 can decrease the growth of lung cancer cells through the NF-κB signaling pathway [9]. The ginsenoside Rh2 notably inhibits prostate tumor growth through the suppression of microRNA-4295, which activates CDKN1A [10].In recent studies, our group has shown that the ginsenoside Rk3 (a rare ginsenoside) has obvious inhibitory activity in the non-small-cell lung cancer [11]. However, the anti-esophageal cancer effects and underlying mechanisms of Rk3 remain unclear.

Therefore, the aim of this study was to research the antitumor effects of the ginsenoside Rk3 on esophageal cancer cell lines and to investigate the potential molecular mechanisms by which it activates apoptosis and autophagy both *in vitro* and *in vivo*. These results provide a better understanding of the potential anticancer mechanisms of the ginsenoside Rk3, which may be an effective drug for esophageal cancer therapy.

## Materials and methods

### Experimental materials

**Fig 1:**
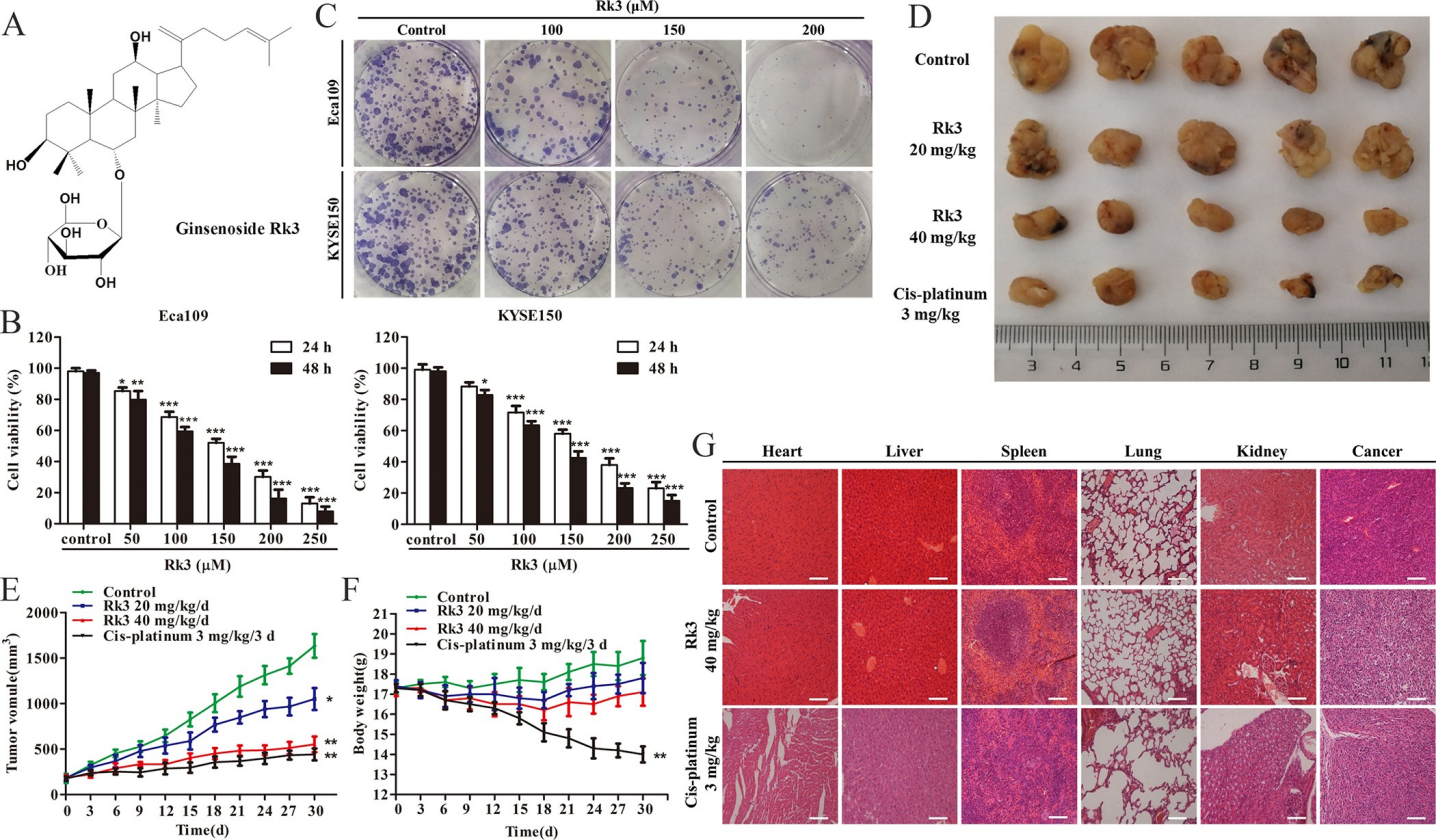
Anti-esophageal cancer effects of the ginsenoside Rk3 *in vitro* and *in vivo*. (A) The chemical structure of the ginsenoside Rk3. (B) Cell viability was measured by the MTT assay. (C) Colony formation. (D) Representative image of the tumor. (E) Tumor volume. (F) Body weight of mice. (G) H&E staining of major organs and tumors. *p < 0.05, **p < 0.01 and ***p < 0.001 compared with control.

The ginsenoside Rk3 (purity > 98%) was obtained from Purification Technology Development Co., Ltd. (Chengdu, China) (Fig 1A). Cis-platinum injection was purchased from Qilu Pharmaceutical Co., Ltd. (Shandong, China). DMEM and RPMI-1640 were purchased from HyClone (LA, USA). Fetal bovine serum (FBS) was obtained from Biological Industries (Kibbuta Beit Haemek, Israel). The inhibitors, 3-methyladenine (3-MA), GSK690693 and rapamycin were obtained from Med Chem Express (NJ, USA). Primary antibodies against GAPDH (Cat# 10494-1-AP), Bad (Cat# 10435-1-AP), Bax (Cat# 50599-2-Ig), Bcl-2 (Cat# 12789-1-AP), caspase-9 (Cat# 10380-1-AP), Atg5 (Cat# 10181-2-AP), Beclin1 (Cat# 11306-1-AP), CDK4 (Cat# 11026-1-AP), p53 (Cat# 10442-1-AP) and p21 (Cat# 10355-1-AP) were purchased from Proteintech Group Inc. (Chicago, USA). Antibodies against cleaved PARP (Cat# ab2317), cytochrome-c (Cat# ab18738), mTOR (Cat# ab2732), p-mTOR (Cat# ab109268) and LC3B (Cat# ab51520) were purchased from Abcam (Cambridge, UK). PI3K (Cat# ABP52199) and p-PI3K (Cat# ABP50495) antibodies were purchased from Abbkine (California, USA). Primary antibodies against SQSTM1/P62 (Cat# 5114), cleaved caspase-3 (Cat# 9664), cyclinD1 (Cat# 2922), Akt (Cat# 9272) and p-Akt (Cat# 4058) were purchased from Cell Signaling Technology (Danvers, USA).

### Cell culture

Eca109 and KYSE150 cells were purchased from ATCC (VA, USA). Eca109 cells were cultured in DMEM, and KYSE150 cells were cultured in RPMI-1640 medium contained with 10% FBS and 1% penicillin-streptomycin. All cell lines were cultured at 37°C in a humidified incubator with 5% carbon dioxide and 95% air.

### MTT assay

Cell viability was measured by MTT assay. Eca109 and KYSE150 cells were cultured in 96-well plates after plating at a density of 8×10^3^ cells per well. After treatment with 0.1% DMSO (control) or Rk3 (50, 100, 150, 200 and 250 μM) for 24 or 48 h, the cells were incubated with 50 μL of 5 mg/mL MTT solution for 4 h. Finally, the supernatant was removed, and 150 μL DMSO was added to dissolve the formazan crystals. The absorbance at 490 nm was read with a microplate reader (Power Wave XS2, Bio-tek Instruments Inc., USA).

### Colony formation assay

Eca109 and KYSE150 cells were grown in 6-well plates after plating at a density of 1000 cells per well. Next, the cells were treated with 0.1% DMSO (control) or Rk3 (100, 150 and 200 μM). The cells were cultured for approximately two weeks until visible colonies formed. The medium was changed every three days. At the end of the experiment, the colonies were fixed with methanol and stained with Giemsa stain (Xi’an, China). The number of colonies continuing more than 50 cells was determined using an inverted microscope.

### Human esophageal cancer xenograft nude mouse model

Four-week-old female BALB-c nude mice (14 ± 2 g) were purchased from Hunan SJA Lab Animal Co., Ltd. (Hunan, China). The mice were housed under specific pathogen-free (SPF) conditions and were provided experimental mouse maintenance feed purchased from Chengdu Dashuo Experimental Animal Co., Ltd. (Sichuan, China) and Milli-Q water.

After acclimation of the mice for approximately one week, KYSE150 cells (2 × 10^7^ cells per mouse) were inoculated into the left flank of the mice. After the tumor volume reached approximately 180 mm3, the nude mice were randomly assigned to four groups (n = 5): the solvent group: mice were injected intraperitoneally (i.p.) with solvent daily; two Rk3 groups: mice were injected with 20 mg/kg or 40 mg/kg Rk3 daily; and the positive control group (cis-platinum group): mice were injected with 3 mg/kg cis-platinum every three days. The injection solvent was saline containing 1% Tween-80. The tumor size was calculated as length × width^2^ / 2. After one month, the mice were sacrificed, and the tumors and vital organs were removed and stored in liquid nitrogen or fixed in formalin for subsequent tests.

This study was carried out in strict accordance with the Animal Ethics Procedures and Guidelines of the People’s Republic of China. The protocol was approved by the Northwest University Animal Ethics Committee (NWU-AWC-20190301M). At the end of the experiment, the mice were euthanized by intraperitoneal injection with an overdose of sodium pentobarbital, and all efforts were made to minimize suffering.

### Histopathology and immunohistochemistry

Tumor tissues and organs, including the heart, lung, spleen, kidney and liver, were fixed in 10% buffered formalin. Then, paraffin-embedded specimens were cut into serial sections (4 mm thick) and stained with hematoxylin and eosin (H&E). Tumor tissues were immunostained with cleaved caspase-3 (1:100), LC3B (1:100), p-AKT (1:50) and p-mTOR (1:50). Immunohistochemistry signals were visualized with DAB, and hematoxylin was used as a counterstain. Images were captured under a Nikon TE 2000 fluorescence microscope.

### Cell cycle analysis

Eca109 and KYSE150 cells were plated in 6-well plates at a density of 4×10^5^ cells/well and then exposed to Rk3 (0, 100, 150 and 200 μM) for 24 h. The cells were digested with trypsin, centrifuged, washed, and then fixed in 70% ethanol at 4°C overnight. The supernatant was removed after centrifugation, and the cells were resuspended in 500 μL RNase A, stained with 5 μL PI, and incubated in the dark for 0.5 h. The cell cycle distribution was determined by flow cytometry (BD Bioscience, Shanghai, China).

### Hoechst 33342 staining assay

Eca109 and KYSE150 cells were plated in 6-well plates and exposed to 150 μM Rk3 for 24 h. Then, the cells were incubated with 100 μL/well Hoechst 33342 (Beyotime Biotechnology, Shanghai, China) at 37°C for 0.5 h. The cells were observed under a fluorescence microscope (Nikon, Tokyo, Japan).

### Quantification of apoptosis by flow cytometry

Eca109 and KYSE150 cells were incubated as described above (cell cycle analysis). Then, the cells were collected and resuspended in 0.5 mL binding buffer. The samples were incubated with 5 μL FITC-Annexin V and 5 μL PI (Xi’an, China) for 30 min at 37°C in the dark. Finally, the fraction of stained cells indicating the percentage of apoptotic cells was detected by flow cytometry.

### Transmission electronic microscopy (TEM)

Eca109 and KYSE150 cells were treated with 0.1% DMSO or 150 μM Rk3 for 24 h. The cells were collected by centrifugation, fixed in glutaraldehyde overnight at 4°C, dehydrated with an alcohol series, embedded in epoxy resin, and sectioned. The ultrathin sections (50 nm) were cut with an ultramicrotome and double stained with uranyl acetate and lead citrate. The ultrastructure of autophagosomes was observed under TEM (JEM-1230, Japan).

### Western blotting

Cell samples and tumor tissues were lysed on ice in RIPA buffer (Beyotime, Shanghai, China) containing 1% PMSF and 1% phosphorylated inhibitors. The protein concentration in the supernatant was measured using a BCA Protein Assay Kit (Thermo Scientific, Fremont, USA) at 562 nm by microplate reader. Total (20 μg) protein was separated by SDS-PAGE and transferred to PVDF membranes. Then, the membranes were incubated with primary antibodies overnight at 4°C, followed by incubation with HRP-linked secondary antibodies and detection by enhanced chemiluminescence (ECL) substrate (Merck Millipore, Massachusetts, USA) with a Gel Image system (Tanon 5200, Shanghai, China). Band intensities were quantified using the Gel Image system.

### Statistical analysis

All data were analyzed by ANOVA using SPSS version 20.0. The results are presented as the mean ± standard deviation (SD) obtained from three independent samples. Differences with *p* < 0.05 (*) were regarded as statistically significant.

## Results

### Anti-esophageal effects of the ginsenoside Rk3 *in vitro* and *in vivo*

The Eca109 and KYSE150 cell lines, which are highly differentiated and poorly differentiated esophageal squamous carcinoma cell lines, respectively, were chosen to further explore the effect of Rk3 on the proliferation of esophageal cancer cells. As shown in Fig 1B, treatment with varying concentrations of Rk3 (0–250 μM) for 24 h or 48 h decreased the viability of both Eca109 and KYSE150 cells in a dose- and time-dependent manner. Especially, treatment with 200 μM Rk3 for 48 h led to 16.2 ± 3.5% Eca109 cell viability. The same findings were observed in KYSE150 cells treated with 200 μM Rk3 (23.2 ± 2.1%). Moreover, as shown in Fig 1C, the number of colony formed by esophageal cancer cells obviously decreased with increasing of Rk3 concentration.

To validate the effects of Rk3 *in vivo*, an esophageal cancer xenograft model was constructed with KYSE150 cells. The morphological changes in the tumors after one month of treatment are shown in Fig 1D. Tumor volume and body weight were measured every three days. Mice in the 20 and 40 mg/kg Rk3 and cis-platinum groups exhibited smaller tumor volumes than those in the solvent group, with inhibition rates of 32.6 ± 6.3%, 66.2 ± 8.4% and 72.8 ± 7.1%, respectively (Fig 1E). It is worth noting that the body weight of both the low-dose Rk3 group (17.80 ± 0.75 g) and the high-dose Rk3 group (17.10 ± 0.68 g) was not significantly lower than that of the solvent group, whereas the mice in the cis-platinum group weighed less (14.13 ± 0.41 g) than those in control group (18.80 ± 0.85 g) (Fig 1F), indicating that Rk3 exhibited less toxicity than cis-platinum. H&E staining of tumor tissue and major organs results confirmed this conclusion. The tumor cells in the solvent control group were arranged tightly and showed deep staining and less intercellular substance. However, the tumor cells in the treatment groups showed a significant reduction in number, lighter staining and partial degeneration (Fig 1G). Meanwhile, histological analysis of the heart, liver, spleen, lungs and kidneys in the 40 mg/kg Rk3-treated group showed no obvious injury compared with the control group, whereas the cis-platinum group showed distinct injury in the structure of major organs, especially liver and spleen cells morphology changes, enlargement of the glomerulus, and collapse of alveoli (Fig 1G). These results suggest that Rk3 dramatically inhibits the growth of esophageal cancer *in vitro* and *in vivo*.

### Rk3 induced cell cycle arrest in Eca109 and KYSE150 cells

**Fig 2:**
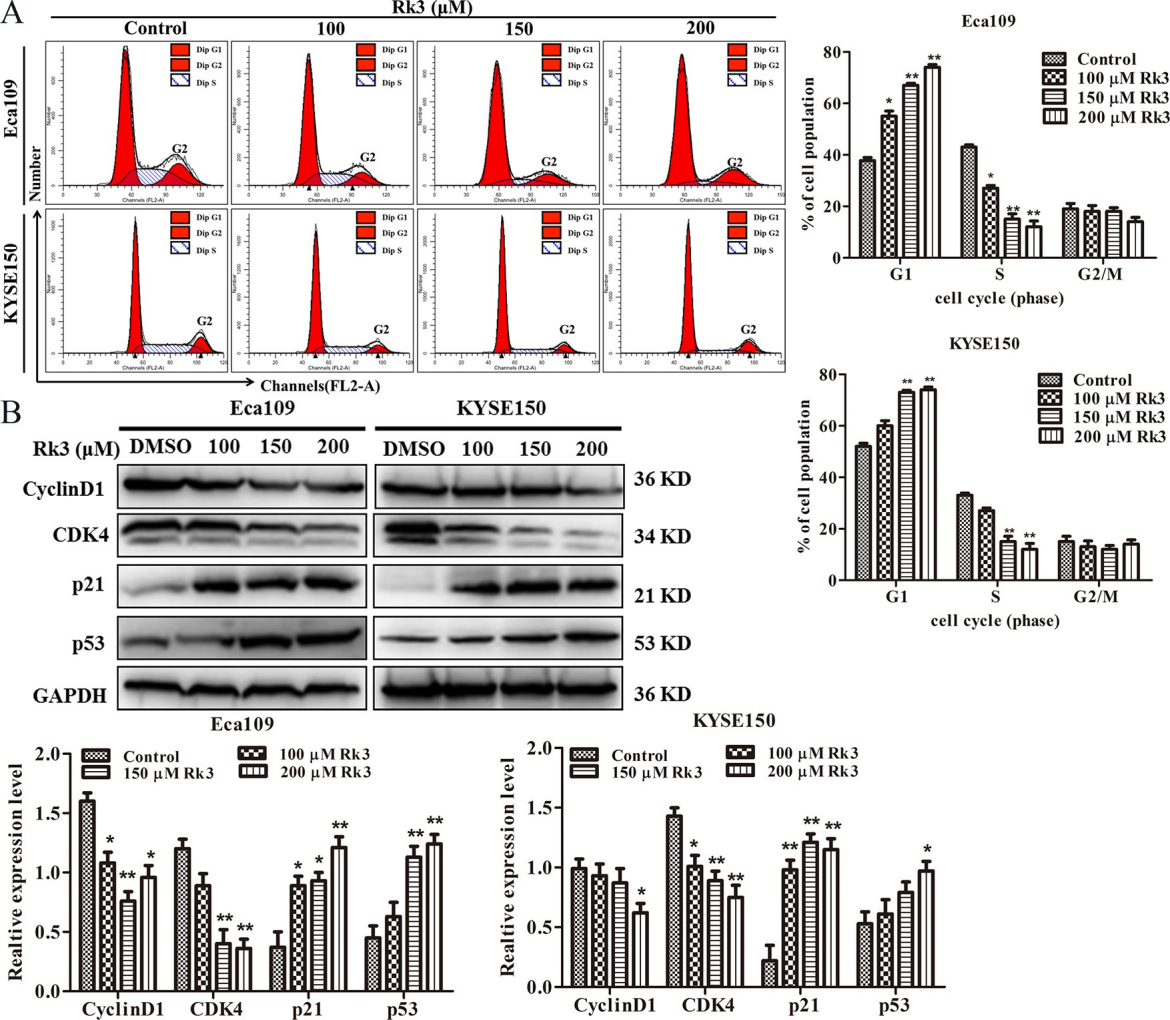
Rk3 triggers G1 arrest in Eca109 and KYSE150 cells. (A) Cell cycle distribution analyzed by flow cytometry. (B) The expression level of G1 transition-related proteins was evaluated by western blotting. *p < 0.05, **p < 0.01 compared with control.

Next, we explored the anti-esophageal cancer mechanism of Rk3. First, flow cytometry was used to investigate which cell cycle phase or checkpoint was affected by Rk3. As shown in Fig 2A, after treatment with 200 μM Rk3, the proportion of Eca109 cells in G1 phase increased from 37.79% to 74.12%, the number of cells in S phase decreased from 43.07% to 12.43%, and the ratio of cells in G2 phase was essentially the same. Similarly, in contrast to the control, treatment with 200 μM Rk3 resulted in an obvious increase (30.05%) in the population of KYSE150 cells in G1 phase and a marked decrease (18.07%) in S phase, the percentage of cells in G2 phase was essentially the same. Next, we detected the expression of G1 cell cycle-related proteins by western blotting. The results displayed that Rk3 dramatically upregulated the expression of p53 and p21, but downregulated the levels of cyclin D1 and CDK4 in a dose-dependent manner in both Eca109 and KYSE150 cells (Fig 2B). Taken together, those findings suggest that Rk3 triggers cell cycle arrest by influencing the levels of proteins involved in the G1 transition.

### Rk3 induced apoptosis in Eca109 and KYSE150 cells

**Fig 3:**
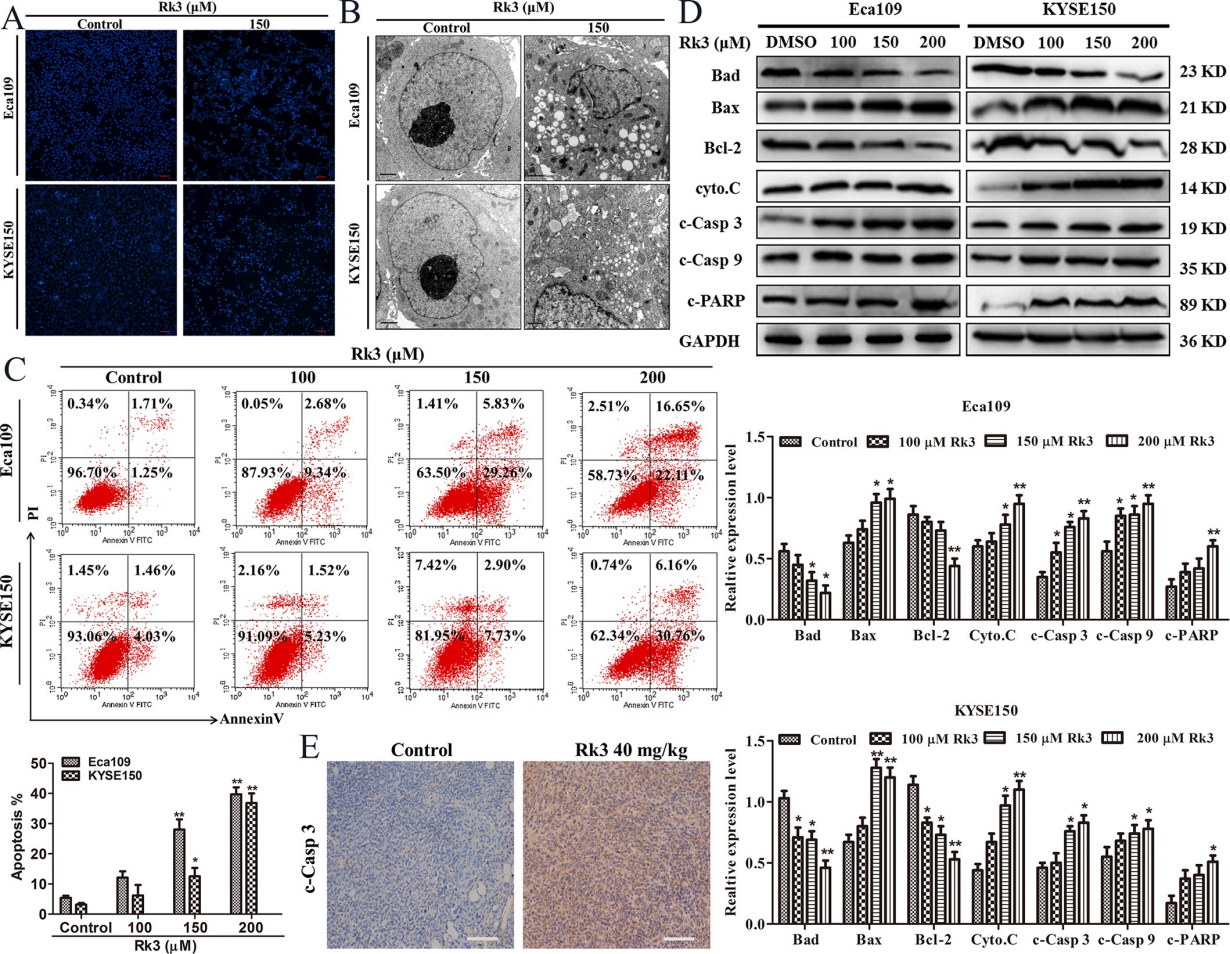
Rk3 induces apoptosis in Eca109 and KYSE150 cells. (A) Hoechst 33342 staining. Scale bar: 100 μm. (B) Ultrastructure of apoptotic cells was observed by TEM. Scale bar: 1 μm. (C) Apoptosis was quantified by flow cytometry. (D) Effect of different doses of Rk3 on the expression of apoptosis-related proteins after treatment for 24 h. (E) Immunohistochemistry staining of tumor tissue. *p < 0.05, **p < 0.01 compared with control.

Cell cycle arrest usually induces cell apoptosis [12]. Therefore, Hoechst 33342 staining, TEM and flow cytometry assays were used to investigate whether Rk3 induces apoptosis. Hoechst 33342 staining images revealed that the number of Eca109 and KYSE150 cells decreased and cell morphology changed (Fig 3A). Moreover, TEM results showed that cells treated with 150 μM Rk3 for 24 h presented apoptotic characteristics of cell membrane shrinkage, nuclear fragmentation, chromatin margination and massive vacuolation (Fig 3B). AV/PI double staining results demonstrated that the number of apoptotic Eca109 cells increased to 39.21% after treatment with 200 μM Rk3 for 24 h. Similarly, the percentage of apoptotic KYSE150 cells clocked up 30.12% (Fig 3C). These results indicate that Rk3 can cause apoptotic cell death.

Furthermore, we explored the expression of crucial proteins involved in apoptosis and DNA repair. As shown in Fig 3D, Bax, cleaved caspase-3, cleaved caspase-9, cytochrome C and cleaved PARP levels were increased after treatment with Rk3, whereas Bad and Bcl-2 levels were reduced in a dose-dependent manner in both Eca109 and KYSE150 cells, indicating that Rk3 activated apoptosis in esophageal cancer cells. Immunohistochemistry staining of tumor tissue showed that the area positive for cleaved caspase-3 was larger in the Rk3 group than in the control group (Fig 3E).

### Rk3 induced autophagy in Eca109 and KYSE150 cells

**Fig 4:**
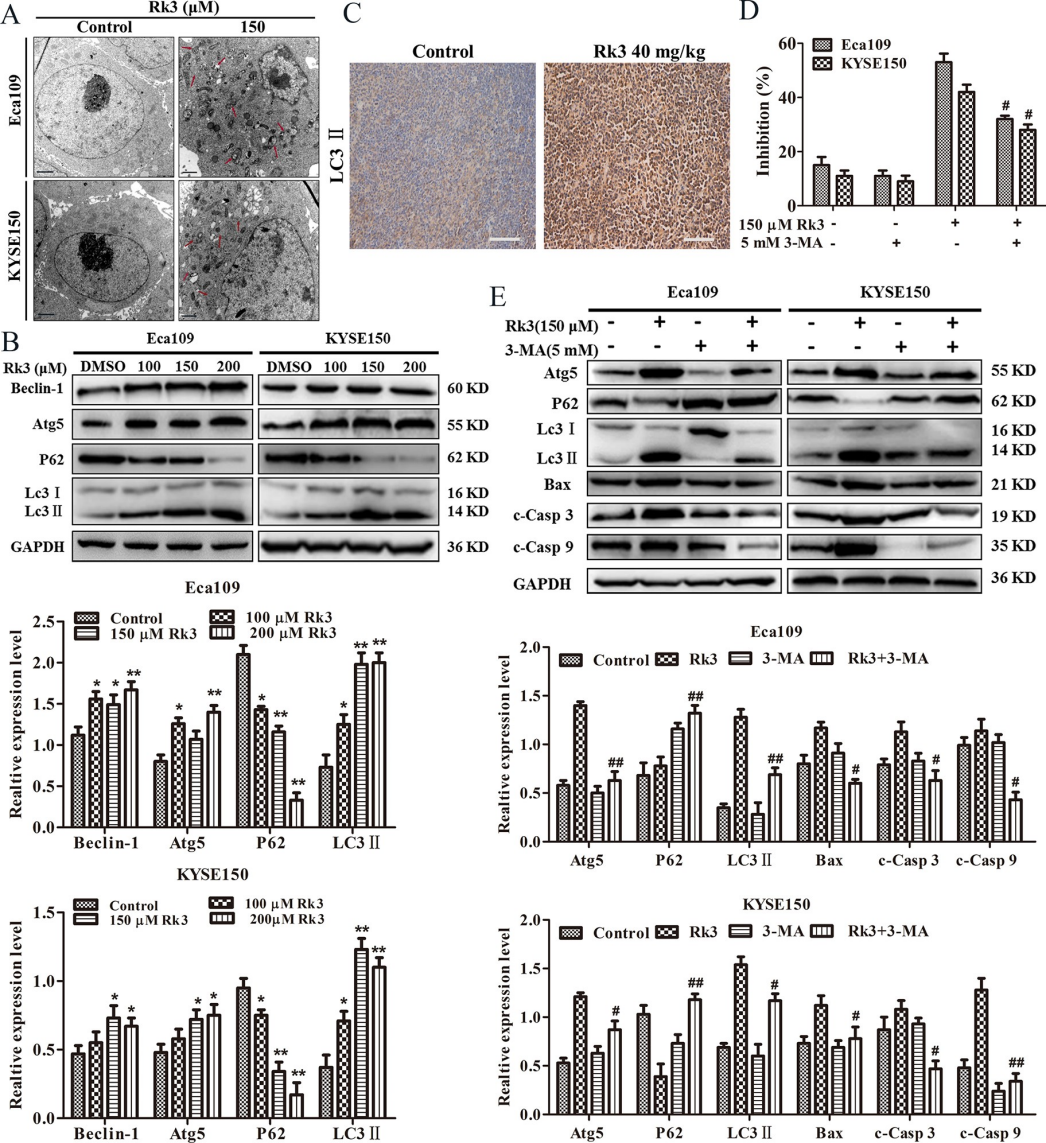
Rk3 induces autophagy in esophageal cancer cells. (A) Autophagosomes were observed under TEM. Red arrows represent autophagosomes (scale bar = 1 μm). (B) Effect of different doses of Rk3 on the expression of autophagy-related proteins after treatment for 24 h. (C) Immunohistochemical staining of tumor tissue. (D) Cells were pretreated with 3-MA (5 mM) for 2 h and then incubated with the indicated dose of Rk3 for 24 h. The inhibition of cell proliferation was measured by MTT assays. (E) Cells were treated as state above, and Atg5, p62, LC3, Bax, c-Casp 3 and c-Casp 9 were analyzed by western blotting. *p < 0.05, **p < 0.01 compared with the control; ^**#**^*p* < 0.05, ^**##**^*p* < 0.01 compared with the Rk3-treated group.

Autophagy is a lysosomal degradation pathway characterized by an increasing quantity of acidic vesicular organelles associated with autophagosomes [13]. To determine whether Rk3 can activate autophagy in esophageal cells, we carried out research on autophagosomes and the relative expression of autophagy-related proteins in Eca109 and KYSE150 cells. As shown in Fig 4A, TEM revealed more autophagosomes in the cytoplasm of Rk3-treated cells than in that of control cells. We also investigated the expression of autophagy-related proteins. Western blotting results were shown in Fig 4B. The level of LC3-II was increased in a dose-dependent manner, and Atg5 and Beclin-1 upregulation and P62 downregulation were also observed in a concentration-dependent manner. Immunohistochemical staining of tumor tissue showed that the LC3-II-positive area was larger in the Rk3 group than in the control group (Fig 4C). As a consequence, our findings indicate that Rk3 triggers autophagy in esophageal cancer cells.

Autophagy can both protect cells from death and reduce cell survival. To determine the effect of autophagy induced by Rk3, the cells were pretreated the autophagy inhibitor 3-MA before Rk3 treatment. The MTT results demonstrated that 3-MA could partially block the inhibitory effect of Rk3 on Eca109 and KYSE150 cell viability, revealing that the autophagy induced by Rk3 was pro-death (Fig 4D). Moreover, western blotting results showed that pretreatment with 3-MA observably attenuated the accumulation of LC3-II; increased the relative expression of Atg5, Bax, cleaved caspase-9 and cleaved caspase-3; and diminished the relative protein expression of P62 (Fig 4E). These findings prove that autophagy induced by Rk3 contributes to cell death and enhances esophageal cell apoptosis.

### Rk3 induced apoptosis and autophagy by blocking the PI3K/Akt/mTOR signaling pathway in esophageal cancer cells

**Fig 5:**
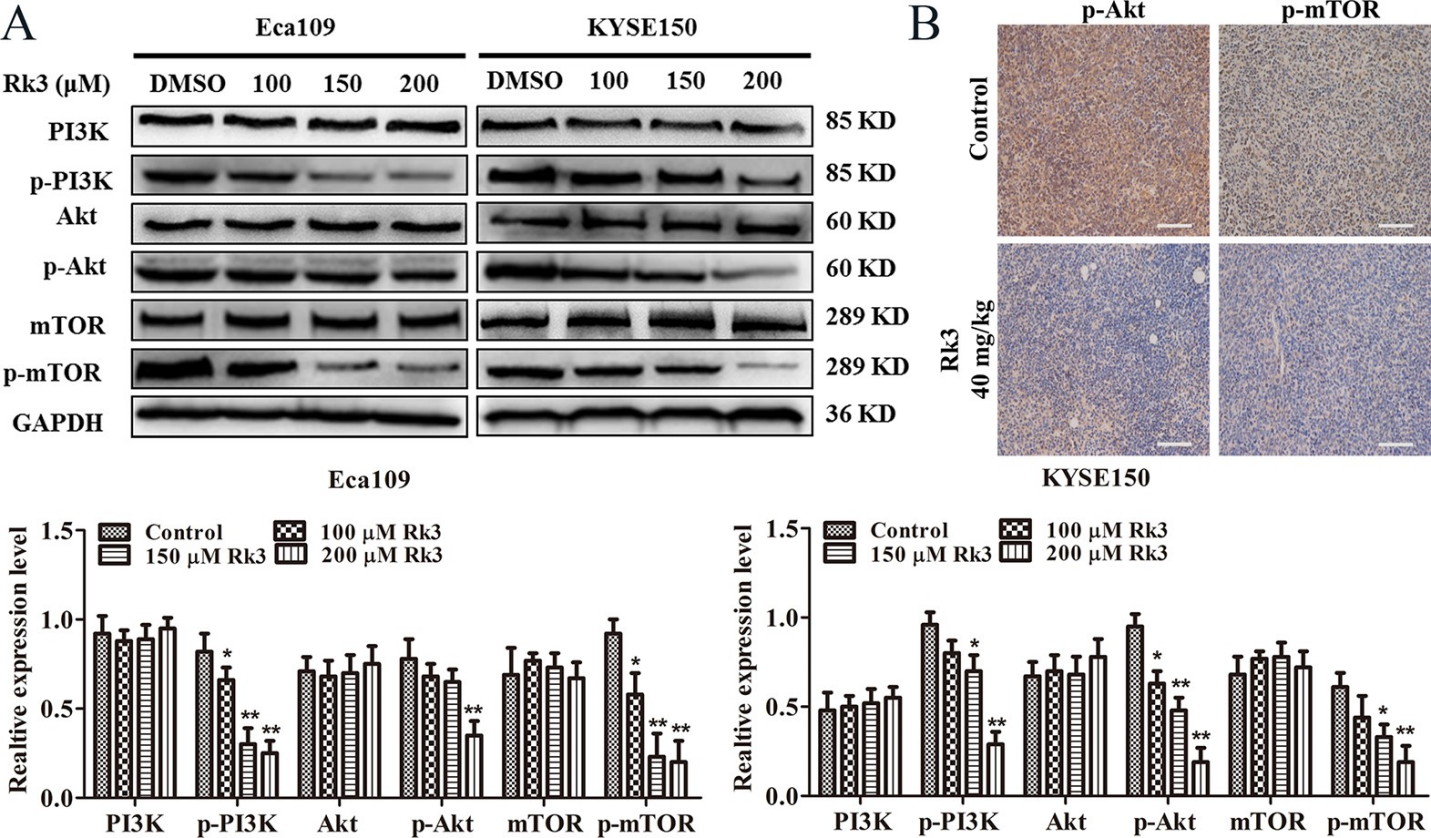
Rk3 suppresses the PI3K/Akt/mTOR pathway in Eca109 and KYSE150 cells. (A) Cells were treated with differernt concentrations of Rk3 for 24 h, and the expression of PI3K, p-PI3K, Akt, p-Akt, mTOR and p-mTOR was determined by western blotting. (B) Immunohistochemical staining of tumor tissue. *p < 0.05, **p < 0.01 compared with the control.

To further understand the molecular mechanism of the anticancer activity of Rk3, we explored the effect of Rk3 on the PI3K/Akt/mTOR pathway. The results showed that Rk3 significantly downregulated the levels of phosphorylated PI3K, Akt and mTOR in a dose-dependent manner but did not change the expression of PI3K, Akt or mTOR in either Eca109 or KYSE150 cells (Fig 5A), suggesting that Rk3 blocked the PI3K/Akt/mTOR pathway. Immunohistochemical staining of tumor tissue showed that the area positive for p-Akt and p-mTOR was smaller in the Rk3 group than in the control group (Fig 5B).

**Fig 6:**
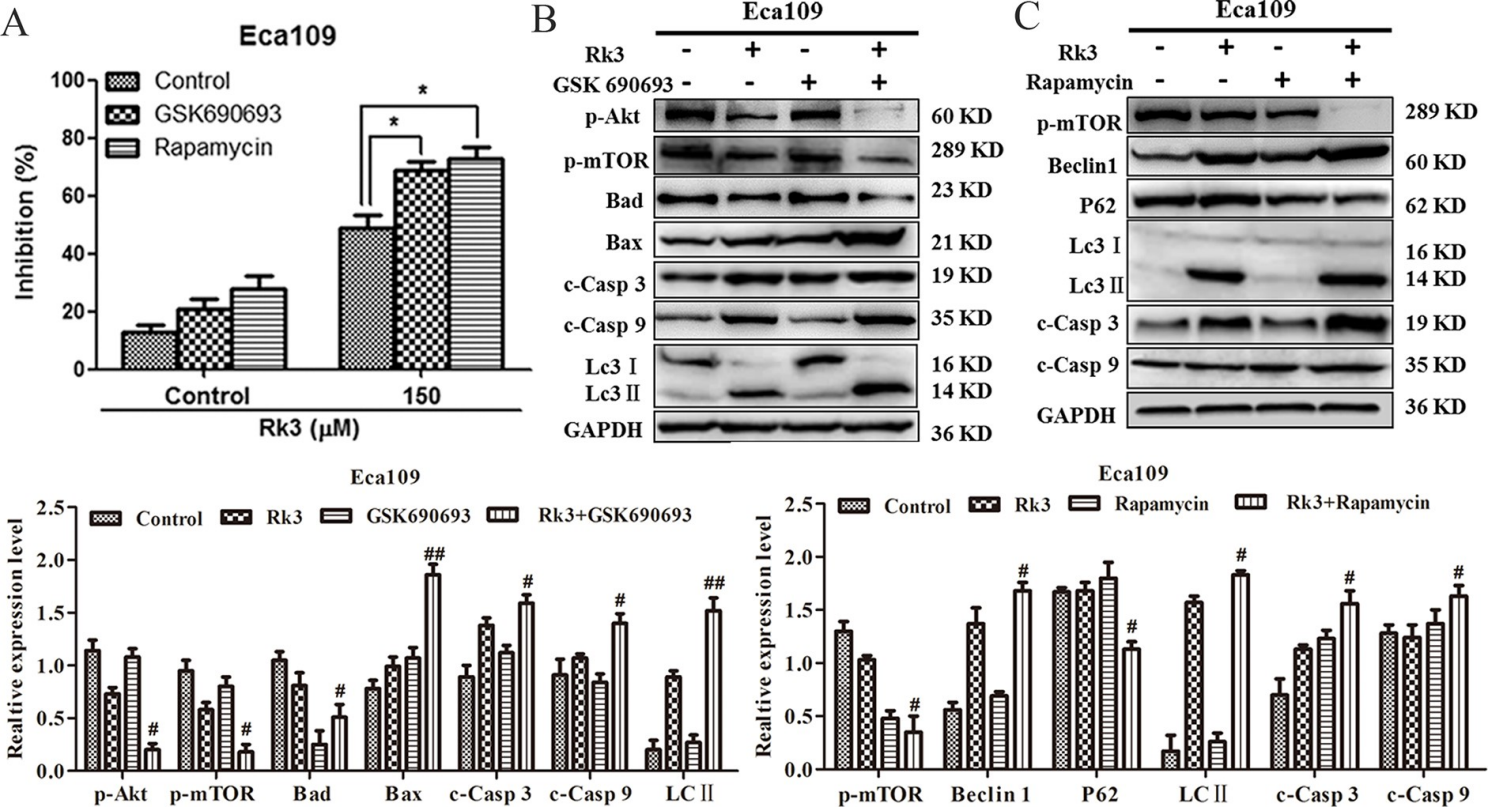
Rk3 induces apoptosis and autophagy by blocking the PI3K/Akt/mTOR pathway in esophageal cancer cells. (A) Cells were pretreated with GSK690693 (25 nM) or rapamycin (50 nM) for 2 h and then incubated with 150 μM Rk3 for 24 h. The inhibition of cell proliferation was detected using MTT assays. (B) and (C) The expression of apoptosis- and autophagy-related proteins was analyzed by western blotting after pretreatment with inhibitors. Cells were treated as stated above. *p < 0.05 compared with the control; ^**#**^*p* < 0.05, ^**##**^*p* < 0.01 compared with the Rk3-treated group.

Next, to verify whether Rk3 can induce apoptosis and autophagy through the PI3K/Akt/mTOR pathway, we exposed the cells to GSK690693 (an Akt inhibitor) or rapamycin (a mTOR inhibitor) before treatment with Rk3. Notably, the MTT results showed that the ability of Rk3 to inhibit Eca109 cell growth was further enhanced by both GSK690693 and rapamycin (Fig 6A). Moreover, western blot analysis revealed that pretreatment with GSK690693 blocked the phosphorylation of Akt and mTOR and activated apoptosis and autophagy in Eca109 cells (Fig 6B). Similarly, pretreatment with rapamycin repressed mTOR phosphorylation and activated apoptosis and autophagy in Eca109 cells (Fig 6C). These data show that Rk3 can induce apoptosis and autophagy in esophageal cancer cells through regulation of the PI3K/Akt/mTOR pathway.

## Discussion

Traditional surgery, radiotherapy, and chemotherapy often have many side effects that limit the successful treatment of esophageal cancer. Hence, we urgently seek new therapeutic strategies. The ginsenoside Rk3 is the main bioactive component derived from ginseng and Panax notoginseng. Rk3 was found to have strong anti-lung cancer activities *in vitro* and *in vivo* associated with cell cycle arrest and apoptosis [11]. However, the anticancer efficacy and mechanism of Rk3 in esophageal tumors have not yet been elucidated. This research aimed to explore the anticancer efficacy and molecular mechanisms of Rk3 on esophageal cancer.

In the present study, the MTT and colony formation assays showed that Rk3 had a potent anti-proliferative effect against Eca109 and KYSE150 cells *in vitro*. Moreover, in the esophageal cancer xenograft model, Rk3 significantly inhibited tumor growth. In particular, the inhibition rate in the 40 mg/kg Rk3 group was 66.2%, and there was no significant difference compared with the cis-platinum group (72.8%). In addition, there was no significant difference in the body weight between the Rk3 group and the solvent group, but the body weight of mice administrated cis-platinum was significantly lower. H&E staining of organ tissues from mice treated with cis-platinum showed liver, spleen and kidney injury, demonstrating the high toxicity of this compound, which is consistent with previous studies [14, 15]. In contrast, in the high-dose Rk3 treatment group, H&E staining indicated that Rk3 did not affect the normal function of primary organs. In conclusion, these results demonstrate that Rk3 exhibits strong anti-esophageal cancer activity and low toxicity to normal tissues.

Therefore, we next explored the potential mechanism of the anti-esophageal cancer effect of Rk3, which likely linked to cell cycle arrest, apoptosis and autophagy. Disordered cell cycle regulation is one of the distinguishing features of cancer [16, 17]. Inducing cell cycle arrest may be an effective way for antitumor drugs to suppress tumor progression [18]. Two cell cycle kinase complexes, the CDK4/6-cyclin D and CDK2-cyclin E, cooperate to relieve the inhibition of dynamic transcription complexes containing the retinoblastoma protein Rb and E2F. It is clear that cells enter S phase through continuous phosphorylation of Rb by cyclin D and E [19, 20]. When DNA damage occurs, the protein levels of p53 and p21 increase, thereby regulating downstream the CDK2-cyclin E complex [21]. Our findings showed that Rk3 upregulated the protein levels of p21 and p53 but downregulated cyclinD1 and CDK4 protein expression in esophageal cancer cells, indicating that Rk3 induced G1 arrest in esophageal cells.

Apoptosis is the most universal form of programmed cell death. Apoptosis, a gene-regulated phenomenon, has an indispensable effect on chemotherapy efficacy various cancers [17, 22, 23]. The ginsenosides Rg3, Rh2, Rg5, Rk1 and Rh4 have been proved to trigger apoptosis in multiple types of cancer cells [9, 11, 22, 24–26]. The Bcl-2 protein family, which includes the anti-apoptotic proteins Bcl-2 and Bad and the pro-apoptotic protein Bax, are the crucial initiators of the intrinsic mitochondrial apoptosis pathway [27]. Bax alters the permeability of the mitochondrial membrane to release cytochrome-c from the mitochondrial intramembrane space into the cytoplasm, which led to the activation of caspase-9 and the subsequent activation of caspase-3, ultimately resulting in the cleavage of PARP proteins, which activates the intrinsic apoptosis pathway [28]. In this study, Rk3 induced apoptosis in esophageal cancer cells through activation of Bax, cytochrome-C, cleaved caspase-3, cleaved caspase-9 and PARP and decreased the protein expression levels of Bad and Bcl-2, indicating that Rk3 induced apoptosis by triggering the intrinsic mitochondrial pathway in esophageal cells.

In addition to apoptosis, autophagy also has a deterministic impact on the fate of cells, protecting cell survival or promoting apoptosis [29]. Atg5 and Beclin-1 are necessary for the initiation of autophagy and the maturation of autophagosomes [30, 31]. LC3B-II, a crucial marker of autophagy, is produced from LC3B-I during the formation of autolysosomes [32]. P62 is a typical negative regulator of autophagy that can promote the movement of ubiquitinated substrates to autophagosomes [33]. Previous studies support the results obtained in this study of Rk3-induced autophagy, which was evidenced by the upregulation of Beclin1, LC3-II and Atg5 and downregulation of P62. Accumulating evidence indicates that autophagy plays a dual role in protecting cell survival and contributing to cell death in cancer [34, 35]. Apoptosis and autophagy are interconnected by nodes of multiple molecular crosstalk, and their joint regulation affects the tumor suppressive pathway [36, 37]. Further experiments showed that 3-MA weakened the inhibitory effect of Rk3 on esophageal cells, suggesting that Rk3 induced-autophagy likely contributes to cell death. Western blotting results showed that apoptosis was inhibited following the inhibition of autophagy in Eca109 and KYSE150 cells. These results clarify that autophagy induced by Rk3 can promote apoptosis.

The PI3K/Akt/mTOR pathway is a crucial signaling cascade that is activated in various cancers, and this pathway is associated with cell proliferation, invasion, and migration [23, 38]. The PI3K/Akt/mTOR signaling pathway can negatively regulate autophagy by mediating p-mTOR levels. The phosphorylation of Akt is a significant event in the apoptosis process [25, 39–41]. Moreover, Bad is considered the center of the pro-apoptotic and anti-apoptotic regulatory cascades and can directly contact with the PI3K/Akt pathway during apoptotic pathway [42, 43]. In this work, we showed that Rk3 repressed the PI3K/Akt/mTOR pathway. Then we further investigated the effects of this signaling pathway on the crosstalk between apoptosis and autophagy induced by Rk3. The results showed that the suppression of p-Akt with GSK690693 dramatically increased Bax, cleaved caspase-9, cleaved caspase-3 and LC3-II levels and reduced Bad and p-mTOR levels in Eca109 cells. In addition, rapamycin markedly enhanced the levels of Beclin1, LC3-II, cleaved caspase-3 and cleaved caspase -9 and downregulated P62 and p-mTOR. Taken together, these results suggest that Rk3 can trigger apoptosis and autophagy in esophageal cancer through regulation of the PI3K/Akt/mTOR signaling pathway.

## Conclusions

**Fig 7:**
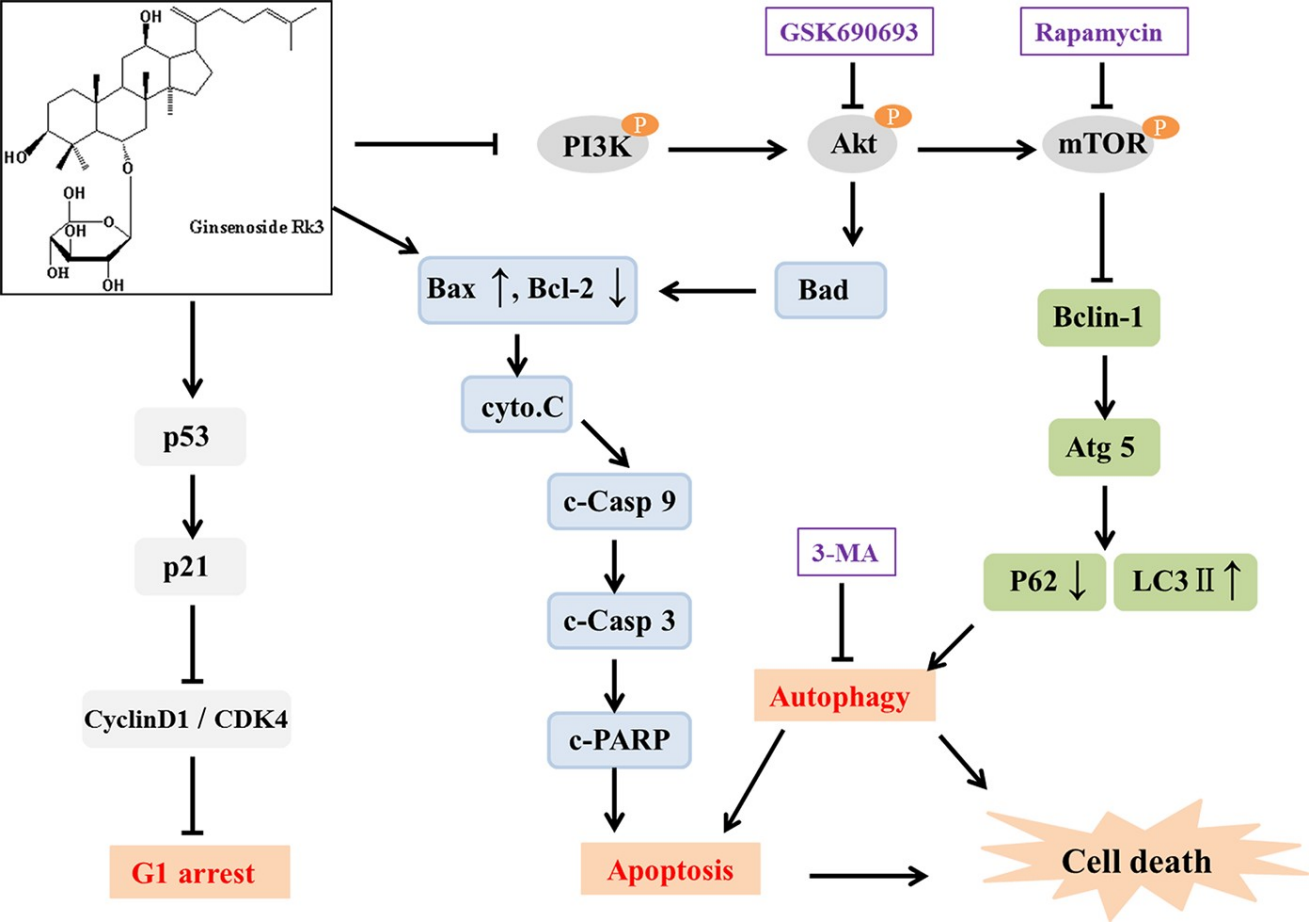
Proposed molecular mechanism of the anti-esophageal cancer activity of Rk3. The PI3K/Akt/mTOR signaling pathway is involved in apoptosis and autophagy induced by Rk3 in esophageal cancer.

The results of the present study illustrate that Rk3 has strong anticancer activity in esophageal cancer cells *in vitro* and *in vivo*. Furthermore, Rk3 inhibited cell proliferation, caused G1 arrest, and activated apoptosis and autophagy in Eca109 and KYSE150 cells. Moreover, we discovered that Rk3 contemporaneously induced apoptosis and autophagy by suppressing the PI3K/Akt/mTOR pathway in esophageal cancer cells. In summary, the potential molecular mechanism of Rk3-induced esophageal cancer cell death is shown in Fig 7. The results presented herein show that the ginsenoside Rk3 may be a new effective drug for esophageal cancer therapy.

## Supporting information

S1 Table Effect of ginsenoside Rk3 on the expression levels of G1 cyclin in Eca109 and KYSE150 cells as assessed by western blotting. (DOCX)

S2 Table Effect of ginsenoside Rk3 on the expression levels of apoptotic proteins in Eca109 and KYSE150 cells as assessed by western blotting. (DOCX)

S3 Table Effect of ginsenoside Rk3 on the expression levels of autophagy proteins in Eca109 and KYSE150 cells as assessed by western blotting. (DOCX)

S4 Table Effect of ginsenoside Rk3 on the protein expression levels of Eca109 and KYSE150 cells pretreated with 3-MA as assessed by western blotting. (DOCX)

S5 Table Effect of ginsenoside Rk3 on the protein expression levels of PI3K-Akt- mTOR pathway in Eca109 and KYSE150 cells as assessed by western blotting. (DOCX)

S6 Table Effect of ginsenoside Rk3 on the protein expression levels of Eca109 cells pretreated with GSK690693 as assessed by western blotting. (DOCX)

S7 Table Effect of ginsenoside Rk3 on the protein expression levels of Eca109 cells pretreated with rapamycin as assessed by western blotting. (DOCX)

## Abbreviations

Rk3: ginsenoside Rk3
c-Casp 3: cleaved caspase-3
c-Casp 9: cleaved caspase-9
cyto.C: cytochrome C
c-PARP: cleaved PARP
P62: SQTM1/P62
