# Supplementary material for: The ginsenoside Rk3 exerts anti-esophageal cancer activity in vitro and in vivo by mediating apoptosis and autophagy through regulation of the PI3K/Akt/mTOR pathway
Source: PLoS One. 2019 May 15;14(5):e0216759. doi: 10.1371/journal.pone.0216759 (PMC6519821; doi:10.1371/journal.pone.0216759)
Supplement: S1 Table — (DOCX) [file pone.0216759.s001.docx]

Table 1.Effect of ginsenoside Rk3 on the expression levels of G1 cyclin in Eca109 and KYSE150 cells as assessed by western blotting

|  | | N | CyclinD1 | CDK4 | P21 | P53 |
| --- | --- | --- | --- | --- | --- | --- |
| Eca109 | Control | 3 | 1.60±0.07 | 1.20±0.08 | 0.37±0.13 | 0.45±0.10 |
|  | 100 μM Rk3 | 3 | 1.08±0.09***** | 0.89±0.10 | 0.89±0.08***** | 0.63±0.12 |
|  | 150 μM Rk3 | 3 | 0.76±0.08****** | 0.40±0.12****** | 0.93±0.07***** | 1.13±0.09****** |
|  | 200 μM Rk3 | 3 | 0.96±0.10***** | 0.36±0.08****** | 1.21±0.09****** | 1.24±0.08****** |
| KYSE150 | Control | 3 | 0.99±0.08 | 1.43±0.09 | 0.22±0.11 | 0.53±0.09 |
|  | 100 μM Rk3 | 3 | 0.93±0.09 | 1.01±0.08***** | 0.98±0.07****** | 0.61±0.09 |
|  | 150 μM Rk3 | 3 | 0.87±0.11 | 0.89±0.06****** | 1.21±0.08****** | 0.79±0.10 |
|  | 200 μM Rk3 | 3 | 0.62±0.07***** | 0.75±0.10****** | 1.15±0.06****** | 0.97±0.09***** |

The values in the table represent the average gray values relative to GAPDH**.**

********P*<0.05, *********P*<0.01 compared with the control
