# Supplementary material for: The ginsenoside Rk3 exerts anti-esophageal cancer activity in vitro and in vivo by mediating apoptosis and autophagy through regulation of the PI3K/Akt/mTOR pathway
Source: PLoS One. 2019 May 15;14(5):e0216759. doi: 10.1371/journal.pone.0216759 (PMC6519821; doi:10.1371/journal.pone.0216759)
Supplement: S2 Table — (DOCX) [file pone.0216759.s002.docx]

|  | | N | Bad | Bax | Bcl-2 | cyto.C | c-Casp 3 | c-Casp 9 | c-PARP |
| --- | --- | --- | --- | --- | --- | --- | --- | --- | --- |
| Eca109 | Control | 3 | 0.56±0.06 | 0.63±0.06 | 0.86±0.08 | 0.60±0.05 | 0.35±0.04 | 0.56±0.08 | 0.27±0.06 |
|  | 100 μM Rk3 | 3 | 0.45±0.08 | 0.74±0.07 | 0.80±0.04 | 0.64±0.07 | 0.55±0.08***** | 0.85±0.07***** | 0.39±0.06 |
|  | 150 μM Rk3 | 3 | 0.32±0.07***** | 0.96±0.07***** | 0.73±0.07 | 0.78±0.08***** | 0.76±0.04***** | 0.86±0.07***** | 0.42±0.08 |
|  | 200 μM Rk3 | 3 | 0.22±0.06***** | 0.99±0.08***** | 0.44±0.06****** | 0.95±0.07****** | 0.83±0.06****** | 0.95±0.08****** | 0.60±0.05****** |
| KYSE150 | Control | 3 | 1.03±0.08 | 0.67±0.04 | 1.14±0.07 | 0.44±0.06 | 0.46±0.06 | 0.55±0.10 | 0.17±0.06 |
|  | 100 μM Rk3 | 3 | 0.71±0.06***** | 0.80±0.09 | 0.83±0.05***** | 0.67±0.06 | 0.50±0.08 | 0.68±0.06 | 0.37±0.07 |
|  | 150 μM Rk3 | 3 | 0.69±0.09***** | 1.28±0.05****** | 0.73±0.08***** | 0.97±0.10***** | 0.76±0.06***** | 0.74±0.06***** | 0.40±0.09 |
|  | 200 μM Rk3 | 3 | 0.46±0.07****** | 1.20±0.10****** | 0.53±0.08****** | 1.10±0.08****** | 0.83±0.05***** | 0.78±0.09***** | 0.51±0.06***** |

Table 2.Effect of ginsenoside Rk3 on the expression levels of apoptotic proteins in Eca109 and KYSE150 cells as assessed by western blotting

The values in the table represent the average gray values relative to GAPDH**.**

********P*<0.05, *********P*<0.01 compared with the control
