# Supplementary material for: The ginsenoside Rk3 exerts anti-esophageal cancer activity in vitro and in vivo by mediating apoptosis and autophagy through regulation of the PI3K/Akt/mTOR pathway
Source: PLoS One. 2019 May 15;14(5):e0216759. doi: 10.1371/journal.pone.0216759 (PMC6519821; doi:10.1371/journal.pone.0216759)
Supplement: S3 Table — (DOCX) [file pone.0216759.s003.docx]

Table 3.Effect of ginsenoside Rk3 on the expression levels of autophagy proteins in Eca109 and KYSE150 cells as assessed by western blotting

|  | | N | Beclin1 | Atg5 | P62 | LC3Ⅱ |
| --- | --- | --- | --- | --- | --- | --- |
| Eca109 | Control | 3 | 1.12±0.10 | 0.80±0.08 | 2.1±0.11 | 0.73±0.15 |
|  | 100 μM Rk3 | 3 | 1.56±0.09***** | 1.26±0.07***** | 1.43±0.04***** | 1.25±0.12***** |
|  | 150 μM Rk3 | 3 | 1.49±0.12***** | 1.07±0.10 | 1.16±0.07****** | 1.98±0.14****** |
|  | 200 μM Rk3 | 3 | 1.67±0.10****** | 1.40±0.08****** | 0.33±0.09****** | 2.01±0.12****** |
| KYSE150 | Control | 3 | 0.47±0.06 | 0.48±0.06 | 0.95±0.07 | 0.37±0.09 |
|  | 100 μM Rk3 | 3 | 0.55±0.08 | 0.58±0.07 | 0.75±0.04***** | 0.71±0.07***** |
|  | 150 μM Rk3 | 3 | 0.73±0.09***** | 0.72±0.07***** | 0.34±0.07****** | 1.23±0.08****** |
|  | 200 μM Rk3 | 3 | 0.67±0.06***** | 0.75±0.08***** | 0.17±0.09****** | 1.10±0.07****** |

The values in the table represent the average gray values relative to GAPDH**.**

********P*<0.05, *********P*<0.01 compared with the control
