# Supplementary material for: The ginsenoside Rk3 exerts anti-esophageal cancer activity in vitro and in vivo by mediating apoptosis and autophagy through regulation of the PI3K/Akt/mTOR pathway
Source: PLoS One. 2019 May 15;14(5):e0216759. doi: 10.1371/journal.pone.0216759 (PMC6519821; doi:10.1371/journal.pone.0216759)
Supplement: S4 Table — (DOCX) [file pone.0216759.s004.docx]

Table 4.Effect of ginsenoside Rk3 on the protein expression levels of Eca109 and KYSE150 cells pretreated with 3-MA as assessed by western blotting

|  | | N | Atg5 | P62 | LC3Ⅱ | Bax | c-Casp 3 | c-Casp 9 |
| --- | --- | --- | --- | --- | --- | --- | --- | --- |
| Eca109 | Control | 3 | 0.58±0.05 | 0.68±0.13 | 0.35±0.04 | 0.81±0.09 | 0.79±0.06 | 0.99±0.08 |
|  | 150 μM Rk3 | 3 | 1.40±0.04 | 0.78±0.09 | 1.28±0.08 | 1.17±0.06 | 1.13±0.11 | 1.14±0.12 |
|  | 5 mM 3-MA | 3 | 0.50±0.07 | 1.16±0.06 | 0.28±0.12 | 0.91±0.10 | 0.83±0.08 | 1.02±0.07 |
|  | Rk3+3-MA | 3 | 0.63±0.09**^##^** | 1.32±0.08**^##^** | 0.69±0.07**^##^** | 0.60±0.04**^#^** | 0.63±0.09**^#^** | 0.43±0.08**^#^** |
| #KYSE150 | Control | 3 | 0.53±0.06 | 1.03±0.08 | 0.69±0.05 | 0.73±0.07 | 0.87±0.12 | 0.48±0.09 |
|  | 150 μM Rk3 | 3 | 1.21±0.06 | 0.39±0.11 | 1.54±0.09 | 1.12±0.09 | 1.08±0.09 | 1.28±0.10 |
|  | 5 mM 3-MA | 3 | 0.63±0.07 | 0.73±0.09 | 0.60±0.11 | 0.69±0.07 | 0.93±0.06 | 0.24±0.09 |
|  | Rk3+3-MA | 3 | 0.87±0.12**^#^** | 1.18±0.06**^##^** | 1.17±0.05**^#^** | 0.78±0.13**^#^** | 0.47±0.08**^#^** | 0.34±0.07**^##^** |

The values in the table represent the average gray values relative to GAPDH**.**

^#^*P*<0.05, **^##^***P*<0.01 compared with the 150 μM Rk3
