# Supplementary material for: The ginsenoside Rk3 exerts anti-esophageal cancer activity in vitro and in vivo by mediating apoptosis and autophagy through regulation of the PI3K/Akt/mTOR pathway
Source: PLoS One. 2019 May 15;14(5):e0216759. doi: 10.1371/journal.pone.0216759 (PMC6519821; doi:10.1371/journal.pone.0216759)
Supplement: S5 Table — (DOCX) [file pone.0216759.s005.docx]

Table 5.Effect of ginsenoside Rk3 on the protein expression levels of PI3K-Akt- mTOR pathway in Eca109 and KYSE150 cells as assessed by western blotting

|  | | N | PI3K | p-PI3K | Akt | p-Akt | mTOR | p-mTOR |
| --- | --- | --- | --- | --- | --- | --- | --- | --- |
| Eca109 | Control | 3 | 0.92±0.10 | 0.82±0.08 | 0.71±0.08 | 0.78±0.11 | 0.69±0.15 | 0.92±0.08 |
|  | 100 μM Rk3 | 3 | 0.88±0.06 | 0.66±0.07***** | 0.68±0.09 | 0.68±0.07 | 0.77±0.04 | 0.58±0.12***** |
|  | 150 μM Rk3 | 3 | 0.89±0.08 | 0.31±0.09****** | 0.70±0.10 | 0.65±0.07 | 0.73±0.08 | 0.23±0.13****** |
|  | 200 μM Rk3 | 3 | 0.95±0.06 | 0.25±0.07****** | 0.75±0.10 | 0.35±0.08****** | 0.67±0.09 | 0.20±0.12****** |
| KYSE150 | Control | 3 | 0.47±0.07 | 0.96±0.09 | 0.66±0.06 | 0.95±0.09 | 0.68±0.12 | 0.61±0.07 |
|  | 100 μM Rk3 | 3 | 0.50±0.08 | 0.80±0.08 | 0.72±0.07 | 0.63±0.08***** | 0.77±0.06 | 0.44±0.11 |
|  | 150 μM Rk3 | 3 | 0.52±0.10 | 0.70±0.07***** | 0.68±0.09 | 0.48±0.06****** | 0.78±0.07 | 0.31±0.10***** |
|  | 200 μM Rk3 | 3 | 0.56±0.06 | 0.29±0.08****** | 0.78±0.10 | 0.19±0.07****** | 0.72±0.08 | 0.18±0.09****** |

The values in the table represent the average gray values relative to GAPDH**.**

^*^*P*<0.05, **^**^***P*<0.01 compared with the control
