# Supplementary material for: The ginsenoside Rk3 exerts anti-esophageal cancer activity in vitro and in vivo by mediating apoptosis and autophagy through regulation of the PI3K/Akt/mTOR pathway
Source: PLoS One. 2019 May 15;14(5):e0216759. doi: 10.1371/journal.pone.0216759 (PMC6519821; doi:10.1371/journal.pone.0216759)
Supplement: S6 Table — (DOCX) [file pone.0216759.s006.docx]

|  | | N | p-Akt | p-mTOR | Bad | Bax | c-Casp 3 | c-Casp 9 | LC3Ⅱ |
| --- | --- | --- | --- | --- | --- | --- | --- | --- | --- |
| Eca109 | Control | 3 | 1.14±0.10 | 0.95±0.10 | 1.05±0.08 | 0.78±0.08 | 0.89±0.11 | 0.91±0.15 | 0.20±0.09 |
|  | 150 μM Rk3 | 3 | 0.73±0.06 | 0.58±0.07 | 0.81±0.12 | 0.99±0.09 | 1.38±0.07 | 1.07±0.04 | 0.89±0.06 |
|  | 25 nM GSK690693 | 3 | 1.08±0.08 | 0.80±0.09 | 0.25±0.13 | 1.07±0.10 | 1.12±0.07 | 0.84±0.08 | 0.27±0.07 |
|  | Rk3+GSK690693 | 3 | 0.20±0.06**^#^** | 0.18±0.07**^#^** | 0.51±0.12^#^ | 1.86±0.10**^##^** | 1.59±0.08**^#^** | 1.40±0.09**^#^** | 1.52±0.12^##^ |

Table 6.Effect of ginsenoside Rk3 on the protein expression levels of Eca109 cells pretreated with GSK690693 as assessed by western blotting

The values in the table represent the average gray values relative to GAPDH**.**

^#^*P*<0.05, **^##^***P*<0.01 compared with the 150 μM Rk3
